# Changes in white matter functional networks across late adulthood

**DOI:** 10.3389/fnagi.2023.1204301

**Published:** 2023-06-30

**Authors:** Muwei Li, Yurui Gao, Richard D. Lawless, Lyuan Xu, Yu Zhao, Kurt G. Schilling, Zhaohua Ding, Adam W. Anderson, Bennett A. Landman, John C. Gore

**Affiliations:** ^1^Vanderbilt University Institute of Imaging Science, Vanderbilt University Medical Center, Nashville, TN, United States; ^2^Department of Radiology and Radiological Sciences, Vanderbilt University Medical Center, Nashville, TN, United States; ^3^Department of Biomedical Engineering, Vanderbilt University, Nashville, TN, United States; ^4^Department of Electrical and Computer Engineering, Vanderbilt University, Nashville, TN, United States; ^5^Department of Computer Science, Vanderbilt University, Nashville, TN, United States

**Keywords:** fMRI, resting state, BOLD, normal aging brain, white matter (WM), ICA

## Abstract

**Introduction:**

The aging brain is characterized by decreases in not only neuronal density but also reductions in myelinated white matter (WM) fibers that provide the essential foundation for communication between cortical regions. Age-related degeneration of WM has been previously characterized by histopathology as well as T2 FLAIR and diffusion MRI. Recent studies have consistently shown that BOLD (blood oxygenation level dependent) effects in WM are robustly detectable, are modulated by neural activities, and thus represent a complementary window into the functional organization of the brain. However, there have been no previous systematic studies of whether or how WM BOLD signals vary with normal aging. We therefore performed a comprehensive quantification of WM BOLD signals across scales to evaluate their potential as indicators of functional changes that arise with aging.

**Methods:**

By using spatial independent component analysis (ICA) of BOLD signals acquired in a resting state, WM voxels were grouped into spatially distinct functional units. The functional connectivities (FCs) within and among those units were measured and their relationships with aging were assessed. On a larger spatial scale, a graph was reconstructed based on the pair-wise connectivities among units, modeling the WM as a complex network and producing a set of graph-theoretical metrics.

**Results:**

The spectral powers that reflect the intensities of BOLD signals were found to be significantly affected by aging across more than half of the WM units. The functional connectivities (FCs) within and among those units were found to decrease significantly with aging. We observed a widespread reduction of graph-theoretical metrics, suggesting a decrease in the ability to exchange information between remote WM regions with aging.

**Discussion:**

Our findings converge to support the notion that WM BOLD signals in specific regions, and their interactions with other regions, have the potential to serve as imaging markers of aging.

## Introduction

Aging is a complex and heterogeneous process that is known to affect the brain at structural, biochemical, and molecular levels, which may consequently contribute to cognitive decline (Lee and Kim, 2022). Aging effects in brain are often evident in specific types of magnetic resonance image (MRI) including T1-weighted images which provide volumetric measurements of gray matter (GM) structures. It has been consistently reported that the cerebral cortex becomes smaller and thinner with aging (Salat et al., 2004; Wang et al., 2019; Taubert et al., 2020), potentially reflecting neuronal loss over time. However, the aging brain is characterized by decreases in not only numbers of neurons (Terry et al., 1987) but also their myelinated projections, namely white matter (WM), that provides the essential foundation for transmitting electrophysiological signals between GM (Albert, 1993). Alterations in WM have previously primarily been characterized by histopathological degeneration and are often assessed as hyperintensities in T2 FLAIR and reduced anisotropy of water diffusion in diffusion MRI (Madden et al., 2004, 2009; Yoshita et al., 2006; Liu et al., 2017).

Accompanied by the degeneration of nerve cells and fibers, aging usually involves the decline of various brain functions, which have a strong relationship with changes in the blood oxygenation level-dependent (BOLD) signals that are measured by functional MRI (fMRI). While such signals have been comprehensively studied in GM, whether they reliably arise in WM has been considered controversial, leading to a lack of understanding of whether or in what manner WM functions are influenced by aging processes. However, it is clear from our own and other recent studies that although BOLD effects are weaker in WM, using appropriate detection and analysis methods they are robustly detectable (D’Arcy et al., 2006; Fraser et al., 2012; Ding et al., 2013, 2018; Gawryluk et al., 2014; Peer et al., 2017; Courtemanche et al., 2018; Gore et al., 2019; Li M. et al., 2019), and vary with baseline activity (e.g., as induced by different levels of anesthesia), and alter in response to a stimulus (Wu et al., 2016; Ding et al., 2018; Li et al., 2020; Mishra et al., 2020), supporting their interpretation as indicators of neural activity. In addition, although BOLD effects in GM indirectly reflect the metabolic demands of the electrical and biochemical activity of neurons, there is preliminary evidence that BOLD effects in WM may reflect metabolic processes in glial cells that arise during and after axonal transmission aging brain (Schilling et al., 2022). These processes include upholding resting potentials on cell membranes, such as those found in oligodendrocytes, and providing general support for cellular maintenance, such as myelin upkeep. In addition, the glia are altered in the aging process and changes in WM are known to be associated with loss of brain functions in aging and neurodegenerative diseases (Salas et al., 2020). Moreover, WM BOLD signals measured during a resting state, where no external task or stimulus is present, reflect an intrinsic activity that has been shown to be altered significantly in subjects with neurological or psychiatric disorders (Gao et al., 2020; Huang J. et al., 2020; Lin et al., 2020). Thus there are grounds for postulating that BOLD signals in WM may show changes across the lifespan and potentially provide new insights into functional changes with cerebral aging.

In this study we performed a comprehensive analysis of WM BOLD signals across scales in order to investigate possible changes in the functional organization of the brain with normal aging. According to our previous work, resting-state BOLD signals in WM are similar though weaker to GM, organized in a manner where voxels sharing similar time courses may be grouped into spatially independent components (ICs) (Huang Y. et al., 2020). The temporal synchronizations among specific components can be assessed, possibly revealing important aspects of neural communications and networks. Here we reconstructed a graph based on the pair-wise connectivity among ICs, modeling the WM as a complex network and producing a set of graph-theoretical metrics i.e., cluster coefficients, efficiency, and strength, that can be used to probe the topological properties underlying the network (Wang, 2010). Meanwhile, based on the hierarchical structures of the graph, we grouped ICs into three sub-circuits and then assessed the within-/inter- circuit connectivities. All the above measurements served to characterize macroscopic, system-wide properties of brain communication and were found to decrease in older individuals, suggesting a reduced capacity/efficiency in information exchange therein. In addition, our recent work suggests the frequency contents of WM resting state signals differ in magnitudes and shapes from those in GM, and vary with location across the WM (Li et al., 2021; Li M. et al., 2022). Therefore, on a smaller scale, we also evaluated the power spectra of BOLD signals within each IC and observed a significant relationship between their magnitudes and age in more than half of the ICs. This finding adds to our knowledge about the intensity of BOLD fluctuations in WM during normal aging. Our findings converge to support the notion that neural activities are embedded in WM BOLD signals, and the neural activities in specific WM regions and their interactions with others have the potential to serve as imaging markers of aging.

## Materials and methods

### Dataset

Seven hundred and Seventy healthy (Cognitively normal, CDR = 0) individuals were selected from the OASIS-3 database (LaMontagne et al., 2019). Among them, five hundred and ten who have complete fMRI data and passed the quality control criteria (see the preprocessing section for detail) were analyzed (213 males and 297 females whose ages ranged between 42 and 95 years). Many individuals have longitudinal data but here we use the images acquired on only their first visits. All but three individuals were scanned twice in the single session so we included 1,017 image datasets in total. The imaging protocols are described in detail in a previous report (LaMontagne et al., 2019). Briefly, all images were acquired using Siemens TIM Trio 3T (433 individuals) or Siemens BioGraph mMR PET-MR 3T scanners (77 individuals). Participants were placed in a 20-channel head coil with foam pad stabilizers placed next to the ears to decrease motion. MR imaging included various anatomical and functional sequences, but here only resting state fMRI and T1-weighted images are analyzed. In particular, each resting state session was comprised of two runs of 6 min each, repetition time (TR) = 2,200 ms, echo time (TE) = 27 ms, voxel size = 4 mm isotropic, and the number of volumes = 164. T1-weighted images were acquired using a 3D magnetization-prepared rapid acquisition with gradient echo (MPRAGE), TR = 2,400 ms, TE = 3.16 ms, voxel size = 1 mm isotropic.

### Preprocessing

To first process the data, an automated high-performance pipeline was created. Briefly, slice timing and head motion were removed from the fMRI volumes, and then the mean cerebrospinal fluid (CSF) signal and 24 motion-related parameters were modeled as covariates and regressed out from the BOLD signals. The data were then detrended and passed through a temporal filter with a passband frequency of between 0.01 and 0.1 Hz. All of these procedures were carried out using a customized pipeline based on the DPABI toolbox (Yan et al., 2016). The Computational Anatomy Toolbox (CAT12) was then used to segment GM, WM, and CSF tissue based on the T1-weighted images (Gaser et al., 2022). Using co-registration and normalizing functions in SPM12 (Friston, 1994), the filtered fMRI data, along with corresponding tissue masks, were spatially normalized into MNI space (voxel size = 3 × 3 × 3 mm^3^). As the analyses were restricted to WM, a group-wise WM mask was constructed by averaging the WM parcellations (probability maps) that were derived from cat12 across all subjects and applying a threshold. The initial threshold was set to 0.95, which was capable of eliminating effects from GM. However, this cropped out many important WM voxels, particularly small structures spatially located between gray matter regions, e.g., internal and external capsules, that were vulnerable to inter-individual variabilities. We then spatially expanded the WM mask by decreasing the threshold gradually in steps of 0.01 until the overlap between the mask and GM area could be visually noticed on the averaged T1 image (group mean T1). We found that 0.8 was the minimal value that could produce a clean WM mask (Supplementary Figure 1) while retaining most of the important WM structures. After that, the fMRI data within the WM mask were spatially smoothed with a 4-mm full width at half maximum (FWHM) Gaussian kernel. The preprocessed results were subjected to a manual quality control procedure in which the passing criteria included: (1) all the preprocessed results must be successfully generated; (2) the maximal translations and rotations of head motion must be less than 2 mm and 2°, respectively; (3) the mean frame-wise displacement (FD) must be less than 0.5 mm (Power et al., 2014) and (4) the spatial normalization was acceptable by an expert’s visual inspection.

### Group ICA

Spatiotemporal data can be broken down by ICA into spatial ICs, which are considered a basis set that constitutes the original data after an unidentified but linear mixing process. The data in this study were analyzed using the Group ICA of the FMRI Toolbox (GIFT) (Calhoun et al., 2001). Most of the parameters in the toolbox were set to the default values except for the number of ICs and principal components (PCs). Our previous work reliably detected 31 ICs in WM (Huang Y. et al., 2020). To provide as many components as possible to match known functional segmentations, we set the number of ICs to a greater number, 40, in this study. The first step of group ICA is to reduce the temporal dimension of each subject from 164 to 60 (1.5 times the intended number of ICs) using spatial principal component analysis (PCA). Those PCs were then concatenated along their temporal dimensions across all individuals, to produce a signal time course of 1,017*60 dynamics for every voxel. The group data were subjected to PCA once more, with the dimension further decreased from 60 to 40. This produced PCs that accounted for the greatest variations at the group level, and 40 ICs were then estimated using Infomax from these PCs (Bell and Sejnowski, 1995). The spatial map (at the group level) of each IC was rebuilt, translated to z-scores, and thresholded at *z* > 2. Note that the z-score is solely used here for descriptive purposes and has no claimed statistical validity (Mckeown et al., 1998). Finally, the ICs were overlaid back as masks on the fMRI data of each individual to extract averaged time courses of interest, based on which functional networks were constructed by evaluating correlations as discussed below.

### Network measurements

Connectivity matrices were constructed by calculating Pearson’s correlation coefficients between time courses of ICs pair-wise for each subject. Three types of network measurements, including within-IC functional connectivity (FC), inter-IC FC, and graph-theoretical metrics, were extracted and analyzed. Specifically, the within-IC FC, i.e., the average z score obtained from the group ICA served as a measure of functional integrity in each IC. The inter-IC FC is equivalent to Pearson’s correlation between two specific ICs. Five graph-theoretical metrics were calculated using the brain connectivity toolbox (Rubinov and Sporns, 2010), including two global metrics and three local metrics as follows:

(1) global characteristic path length, i.e., the average shortest path length in the network. A shorter path allows for the quicker transfer of information and reduces costs.
(2) global efficiencies, i.e., the average inverse shortest path length in the network. It measures the exchange of information across the entire network.
(3) local cluster coefficients, the fraction of triangular connecting pathways around an IC, equivalent to the fraction of IC’s neighbors that are neighbors of each other. It is a measure of the degree to which ICs in a graph tend to cluster together. This metric has been shown to be useful for understanding the small-worldness of a network.
(4) local efficiency, i.e., the global efficiency computed on IC neighborhoods. It quantifies how well information is exchanged by its neighbors when it is removed.
(5) local strength, the sum of weights of links connected to the IC, often reflecting the influence or centrality of the IC on the network.

Note that all these measurements are calculated based on the weighted FC matrix and only positive weights were preserved for calculation. We then used multiple linear regression to identify which measurements exhibit significant correlations with age. For this, the head motion was parameterized by the framewise displacement (FD) (Power et al., 2014) derived from preprocessing, so that for each measurement *M* we fit the following:


$$M=c\,o\,n\,s\,t\,a\,n\,t+b\,1\times a\,g\,e+b\,2\times a\,g\,e^{2}+b\,3\times g\,e\,n\,d\,e\,r$$

+b⁢4×h⁢e⁢a⁢d⁢m⁢o⁢t⁢i⁢o⁢n+ϵ

As this study focuses on age, this formula can help regress out the effects of gender and head motions by subtracting the terms corresponding to gender and head motion from *M*, producing an *adjusted M*. Theoretically, this adjusted measurement reflects the change due only to age, resulting in a more accurate measure of the correlation between *M* and age. Here *M* can be any measurement obtained in this study, such as within-IC FC, inter-IC FC, graph-theoretical metrics, and the power spectra profiles.

As the data analyzed were acquired from two different scanners, the observed correlation between age and imaging measurements could in theory be attributed to a scanner effect instead of biological changes due to aging. To rule out such an effect, we performed an experiment in which the relationship between age and within-IC FC was evaluated based on data from only one scanner (TIM Trio 3T).

### Sub-circuits and their reorganizations

By applying the Louvain community detection algorithm (Blondel et al., 2008) to the connectivity matrix, the ICs that are tightly connected with each other can be grouped into a community which represents an integrated circuit or network. To evaluate the possible reorganization of circuits with aging, we first divided the subjects into subgroups at 10-year intervals, and then applied the Louvain approach to the average connectivity matrix regarding the youngest group (40–50 years) to produce a baseline circuit configuration. The within- and inter-circuits connectivities were captured from different age groups but based on the same baseline configuration, and then were compared among different age groups. Meanwhile, distinct circuit configurations were separately calculated using the Louvain approach from different age groups and were compared in terms of the memberships of each IC to the circuits.

### Calculation of power spectra

Spectral analysis of signals represents a complementary approach to identifying features of interest, and BOLD effects that appear to be random over intervals may reflect a distinct pattern of component frequencies. We used Fourier transforms (Welch method) (Welch, 1967) to estimate the power spectra of the BOLD time courses of each voxel. Each IC-specific power spectrum was calculated by averaging the power spectra across all voxels therein. The mean powers across the low-frequency band (0.01–0.1) were measured to indicate the intensity of BOLD fluctuations, and their relationships to age were determined using the same regression model as for the network measurements.

## Results

### Relationship between within-IC FC and age

**FIGURE 1:**
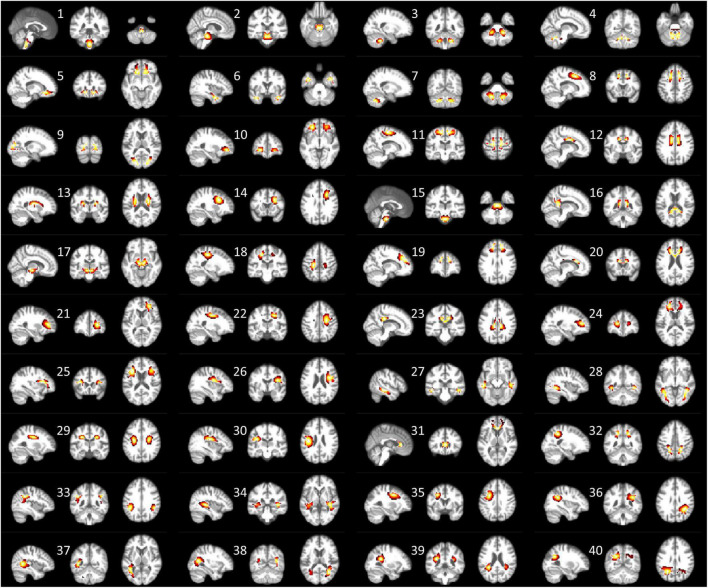
Forty ICs that are derived from group ICA are displayed in three orthogonal planes. The color map overlaid represents the Z score, reflecting the degree of membership of the voxel to the IC. The brighter color indicates a higher Z value. Here only voxels with *Z* > 2 are displayed.

**FIGURE 2:**
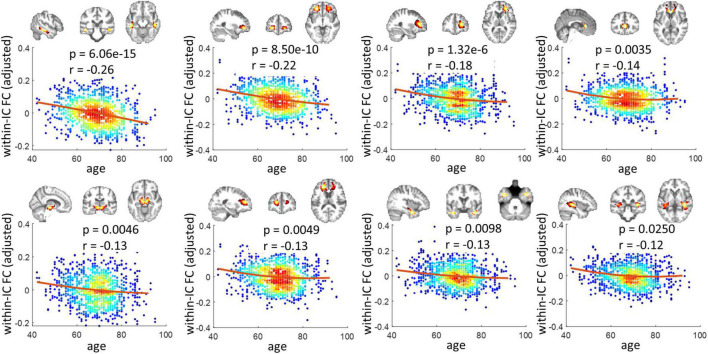
Relationship between within-IC FC and age. Eight ICs that show significant changes across age are shown (*p* < 0.05, Bonferroni correction). Each panel visualizes the spatial distribution of the IC, as well as scatter plots that represent the age of the subjects (x-axis) versus the within-IC FCs (y-axis). Note that the y-axis does not represent the raw FC values but the residues after gender and head motions are regressed out. The color reflects the density of the scatters. The hotter color indicates a higher density.

Figure 1 shows the 40 ICs estimated by the group ICA approach from the resting-state fMRI signals in WM. Each IC was characterized by a cluster of highly connected voxels within a distribution of Z scores representing the voxel-wise FC within the IC. By visual inspection, none of them represents obvious artifacts. These ICs are distributed across the entire WM and show great symmetries between the left and right hemispheres. Some of the ICs reflect known anatomical structures. For example, IC 31, 20, 12, and 16 clearly lay out the genu, anterior body, posterior body, and splenium of the corpus callosum. The corresponding structures of the ICs have been listed in Supplementary Table 1. Each name in the table represents a WM bundle, defined in the JHU WM atlas (Mori et al., 2009), that has the greatest overlap with an IC. By regression, we identified eight ICs whose within-IC FCs varied significantly with age (*p* < 0.05, Bonferroni correction) as shown in Figure 2. Those ICs exhibited reduced within-IC FC in older individuals, and are spatially distributed primarily at the temporal, frontal, and midbrain areas and the genu of the corpus callosum (CC),. From the quadratic fitting of the data, we identified slight decelerations after 70 years old in 5 out of the 8 ICs displayed. Such significant relationships between age and within-IC FCs still exist even if we used the data from a single scanner (Supplementary Figure 2). Therefore it is less likely that the observed age effects on BOLD are attributed to scanner effects.

### Relationship between inter-IC FC and age

**FIGURE 3:**
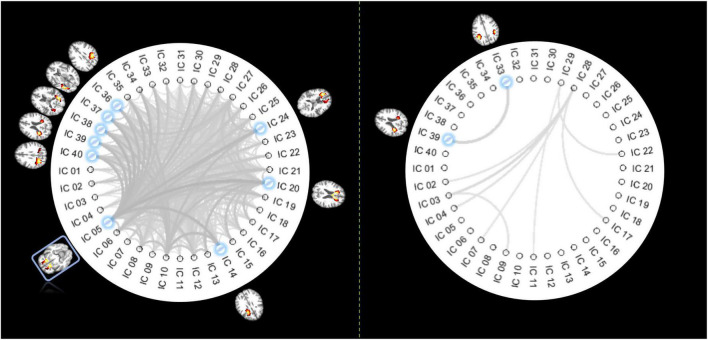
Pair-wise IC connections whose FC show significant correlations with age. The panel on the **left** displays the functional links whose FCs significantly decrease over age (*p* < 0.05, Bonferroni correction). The thicker lines indicate higher r (absolute) values. The ICs involved in the top 8 most significant changes are highlighted in blue, with their distribution maps shown beside the IC labels. Coincidently IC 5 is involved in all those 8 connections of interest so that it is highlighted with a blue rectangle. The panel on the **right** displays the functional links whose FCs significantly increased over age (*p* < 0.05, Bonferroni correction). The thicker lines indicate higher r (absolute) values with age. ICs involved in the most significant changes are highlighted in blue, with their distribution maps shown beside the IC labels.

From 780 possible connections (upper diagonal part of the 40 × 40 FC matrix), we identified 375 pairs of ICs whose FC decreased significantly in older individuals (*p* < 0.05, Bonferroni correction), as shown in the left panel of Figure 3. IC 5, distributed at the inferior frontal area, is involved in the top 8 connections that showed most significant reductions in FC. The other end of those eight connections includes five ICs located at the posterior part of the brain and three ICs at the frontal area (including the genu of the CC). By contrast, there are only 9 connections characterized by increased FC over age (*p* < 0.05, Bonferroni correction), where the most significant change of FC was identified between two IC at the posterior part of the brain.

### Relationship between network metrics and age

**FIGURE 4:**
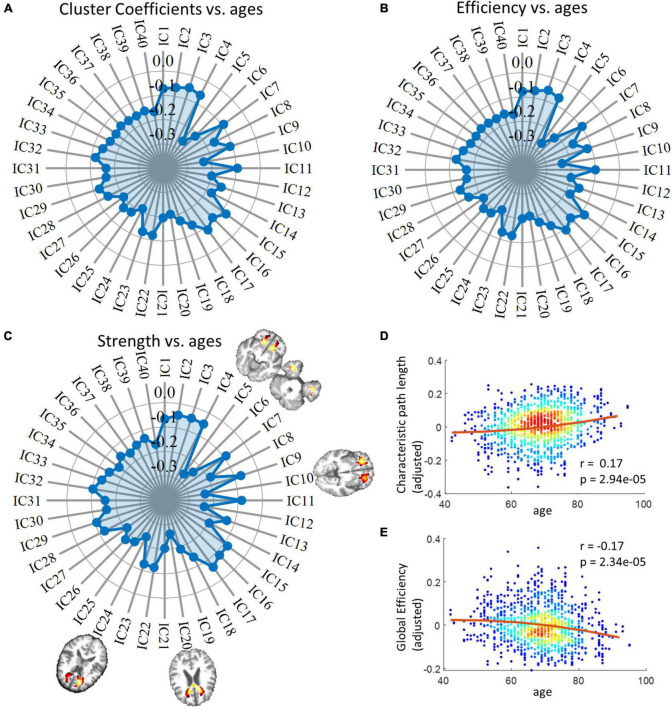
Relationship between network metrics and age. Panel **(A)** displays the relationship between cluster coefficients and age. Each data point on the radar chart indicates r value. Panel **(B)** displays the relationships between network efficiencies and age. Each data point on the radar chart indicates r value. Panel **(C)** displays the relationship between network strength and age. Each data point on the radar chart indicates r value. The distributions of IC 5, 6, 10, 20, and 24, whose spatial distributions are visualized in this panel, are considered IC of interest as they exhibit the closest relationships with age in the case of all three measurements. Panels **(D,E)** display the correlation between global network metrics, including global efficiency and characteristic path length, and age. *P*-values have been corrected by the Bonferroni method. Note that the y-axis does not represent the raw metrics but the residues after gender and head motions are regressed out.

The radar charts in Figure 4 illustrate the relationship between age and three local network metrics, including cluster coefficients, efficiency, and strength. We observed that all forty ICs exhibited reduced metrics over age and the distribution of r values across ICs is in general consistent among the three metrics. For example, the most significant changes are consistently identified in five ICs (highlighted in the Figure) that are distributed in frontal and temporal areas of the brain as well as the genu of the CC. From Figures 4D, E, we observed that the global efficiency of the network decreased significantly whereas the characteristic path length increased significantly over age.

### The reorganization of sub-circuit configurations with age

**FIGURE 5:**
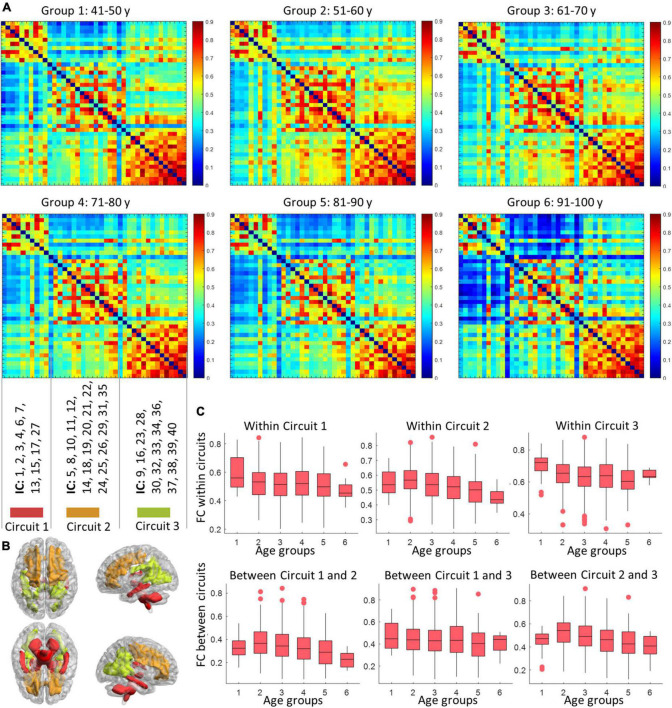
The variation in circuit configuration over age. Panel **(A)** display the mean FC matrices corresponding to six age groups. The nodes (ICs) are sorted according to baseline circuit configuration calculated based on the youngest age group (40–50 years). The distributions of the three circuits are displayed in panel **(B)**, with labels of ICs that are involved in different circuits shown above. Panel **(C)** shows how the within- (first row) and inter- (second row) circuits FC vary with age (groups). Each box visualizes the median, 25, and 75 percentile in the FC values of subjects within an age group.

**FIGURE 6:**
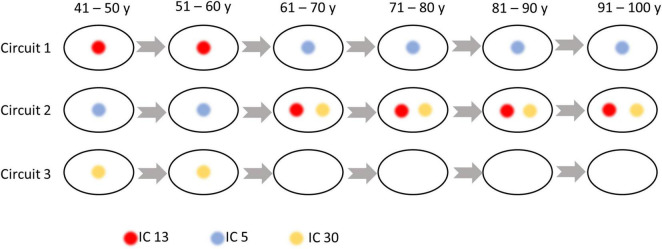
Independent components (ICs) that switch their membership to the circuits over age. IC 13 is a member of circuit 1 but propagates to Circuit 2 at older ages. IC 5 is a member of circuit 2 but propagates to circuit 1 at older ages. IC 30 is a member of circuit 3 but propagates to Circuit 2 at older ages.

As shown in Figure 5, three sub-groups were distinguished by Louvain’s approach, where the first network is composed of ICs at the inferior part of the brain, while the second and third groupings consist of ICs at the anterior and posterior part of the brain. The within- and between-circuit FCs in general decreased in older individuals but were heterogeneous in their trajectories. For example, for FCs in which circuit 2 was involved (within circuit 2, between circuits 1–2 and 2–3), the FC peaked at 50–60 years old. For circuit 3, the within-circuit FC in the 90–100 years group is higher than in some of the younger groups. In terms of circuit propagation, as shown in Figure 6, IC 13 and IC30 are members of circuit 1 and circuit 3, respectively but propagate to circuit 2 at older ages. By contrast, IC 5 is a member of circuit 2 but propagates to circuit 1 at older ages.

### Relationship between power spectra and age

**FIGURE 7:**
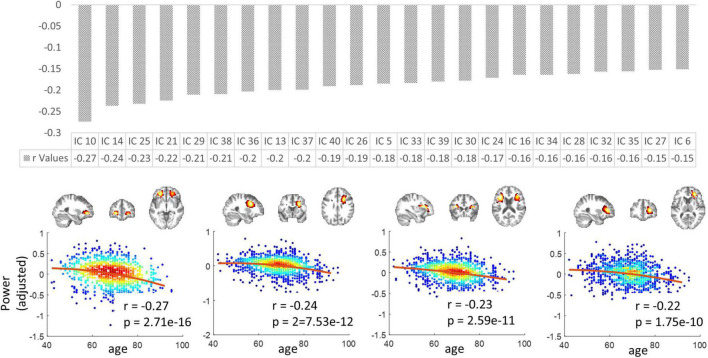
The variation in spectral powers over age. **Upper panel:** the r values (in descending order) corresponding to the 23 ICs in which the mean low-band powers decreased significantly with aging. **Lower panel:** four representatives corresponding to the highest r (absolute) values. Note that the y-axis does not represent the raw power values but the residues after gender and head motions are regressed out. *P*-values have been corrected by the Bonferroni method.

The mean spectral powers decreased significantly with aging in 23 out of 40 ICs (*p* < 0.05, Bonferroni correction). Figure 7 upper panel displays the r values corresponding to those 23 ICs in descending order. In Figure 7 lower panel, we visualize the four representatives corresponding to the highest r (absolute) values. Consistently, these four displayed ICs are all distributed in the frontal areas of the brain.

## Discussion

Following standard methods that have previously been used to analyze BOLD signals in GM, we have modeled WM as a complex network, measured the network properties at different scales, and investigated their correlations to normal aging. We observed that the aging brain exhibits reduced network connections, whether measured locally or globally, suggesting an overall decline in the ability to exchange information between GM regions. In addition, in the majority of WM areas, the spectral power varied significantly with aging, potentially implying changes in the intensities of BOLD fluctuations therein.

Our analysis revealed 40 nodes derived as spatially unique ICs that were identified using a group-ICA approach. The spatial distributions of the ICs are consistent with those in previous works identified based on either ICA (Huang Y. et al., 2020) or K-means clustering (Peer et al., 2017; Li J. et al., 2019, 2022; Wang et al., 2022; Yang et al., 2022). In most of those studies, the nodes that act in concert were further grouped into sub-groups based on their spatial distance to GM, namely, superficial, middle, and deep layers. The intra- and inter-layer assignments were assessed and found to be relevant to specific neurological conditions (Li J. et al., 2022). Though promising, such grouping was determined by anatomic locations and therefore does not reflect any intrinsic functional specialties. By contrast, here we used a data-driven, unsupervised approach to decompose the ICs into functional communities (sub-circuits) by maximizing the within-community connectivities and minimizing the inter-circuits connectivities. Each circuit/community is more likely to represent a distinct function. The temporal interactions between those ICs were mathematically modeled by a graph, producing a set of network metrics, and providing global and local descriptions of the network. The characteristic path length, global efficiency, strength, clustering coefficient, and local efficiency that were quantified in this work represented the measures of connectivity capacity, information exchange at whole-brain and local levels, degree of clustering, and information integration of the functional network, respectively. We observed that ICs in the same functional circuit are in close proximity. But rather than grouped into layers, the three sub-circuits of ICs represent the anterior, posterior, and inferior parts of the brain, and their connectivities, in general, showed a decreasing trend with aging. By contrast, the connectivities within circuit 3 and between circuits 1 and 3 are noticeably higher in the oldest group than those in some younger groups. Similar findings were reported in previous work where the oldest group showed increased connectivity than younger groups (Farràs-Permanyer et al., 2019) and cognitively abnormal individuals showed increased connectivities between temporal and occipital areas (He et al., 2007), possibly due to a compensatory mechanism. This is further confirmed by Figure 3, where the FC between two regions that are located in the posterior brain increased significantly with aging.

The findings regarding the within-IC FC suggest that the frontal and temporal WM regions are more affected by aging. Previous works have reported that age-related changes showed the greatest effects in the frontal lobe, followed by the temporal lobe in many aspects, but predominantly characterized by loss of cortical volumes (Bartzokis et al., 2001; Raz et al., 2005). Other studies have observed reduced WM integrity (O’Sullivan et al., 2001; Gunning-Dixon et al., 2009) in frontal and temporal areas based on diffusion MRI. Therefore, one possible explanation of our finding is that a loss of neurons as well as their myelinated extensions might be associated with lower demand for communications among WM voxels, leading to reduced within-IC FC. We observed a significant reduction of within-IC FC in the genu (anterior part) of CC which connects bilateral frontal regions. This notion is supported by a previous work suggesting age-related decreases in interhemispheric FC between the ventromedial prefrontal cortices (Zhao et al., 2020). Note that in Figure 2, the y-axis represents the adjusted FC measurements in which the individual-wise head motions have been regressed out. The reason for doing this is that older individuals often showed greater head motion during the scan (Supplementary Figure 3 lower panel), which could introduce spurious increases in connectivity (Kato et al., 2021). An interesting finding is that, by contrast, if the head motions are not regressed out from the data, we identified four ICs in which the within-IC FC increased significantly with aging (Supplementary Figure 3), and more importantly, they distributed near the precentral gyrus or within the cerebellum, and thus may be relevant for motor effects. We suspect that the higher neural activities or inter-voxel communication in these regions in older individuals shaped those positive correlations. However, these disappeared (at least were not significant anymore) after the head motions were controlled during regression analysis, suggesting that head motions should be carefully treated when assessing age-related changes in fMRI measurements.

On a larger scale, we observed that nearly half of inter-IC connections exhibit widespread decreases in FC with aging, while only a few, predominantly short-range connections between specific posterior regions, show an increasing trend. Similarly, as reported in previous literature, FC decreased in the connections between most pairs of GM regions but increased only in regions within visual networks which were located at the posterior part of the brain (Zonneveld et al., 2019). A more interesting finding is that an IC at the inferior frontal area is the most affected by aging in terms of a significant reduction of its interactions with the other eight ICs. This finding confirms the vulnerability of the inferior frontal brain to aging and supports the findings reported by Feng et al. (2020) where reduced volume and cerebral blood volume (CBV) were identified. Moreover, the other eight ICs mentioned above show a clear separation: five ICs at the posterior and three ICs at the anterior part of the brain, reflecting two distinct patterns of connections (a short-range and a long-range) that are affected. In addition, the graph metrics indicate a widespread reduction across nearly all regions, among which five frontal ICs appear to be most affected by aging, again confirming the vulnerability of the frontal brain to aging. On an even larger scale, the WM ICs group into three sub-circuits. The FCs within and between the sub-circuits consistently reduced in older individuals, suggesting abnormal communications across all major communities of WM nodes. Such parallel neurodegenerations observed in the anterior, posterior, and inferior parts of the brain WM are consistent with previous findings in which nearly all communities that consisted of GM nodes exhibit negative correlations with age (Varangis et al., 2019). By contrast, different sub-circuits showed different trajectories in their changes in FC over age groups. This can be explained by the distinct time-dependent patterns of changes that have been observed in different regions of the brain (Beason-Held et al., 2008). Moreover, on a global scale, the information exchange, measured by the global metrics, significantly decreases with aging, and the fitting line exhibited a noticeable inflection point at around the 7th decade. This is consistent with the notion that the most notable loss of neurons occurs after 70 years of age (Scahill et al., 2003), possibly leading to less demand for communications between WM regions that are used to mediate neural signal transmission.

Based on our previous work, the local HRF in WM is strongly correlated with the shape of the power spectra of the BOLD signals based on a very short sampling rate (TR = 0.72 s) (Li et al., 2021). However, the data interpreted in the current study are based on a longer TR (2.2 s), and might not provide sufficient temporal resolution to characterize the distribution of power at specific frequencies. Therefore, instead, we measured the mean power across the entire low-frequency band to represent the intensity of BOLD fluctuations, which are also shown to be correlated with resting-state cerebral flow (Zou et al., 2009). The spectral power decreases significantly with aging, particularly in frontal regions. This might be either explained by a reduced demand for signals to be transmitted between neurons or the decreased supply of cerebral blood flow (flow) due to the stiffening and wall thickening of arteries (Tarumi and Zhang, 2018; Rosenberg et al., 2020). Moreover, a previous study suggests that, across studies, the most consistent finding in normal aging is decreased metabolism and cerebral flow in the frontal regions (Xu et al., 2017). However, limited by the temporal resolution of the data, the variation of power over frequencies could not be accurately assessed, leading to a lack of characterization of age-related changes in hemodynamic profiles that were shown to be associated with power spectral shapes. This will be examined in the future using fMRI data acquired based on faster repetition times. Indeed, all the above observations need to be interpreted cautiously as BOLD effects originate from the hemodynamic response to increased demands for energy substrates and are only indirect metrics of neural activity and communication. Age-related changes in microvascular tone and volume may explain some of the effects reported above. Although BOLD signal increases are usually interpreted as physiological responses to increased demands for oxygen that are required for increased metabolism, the nature and driving force for such responses in white matter are unclear. Harris and Attwell (2012) predicted that the major energy budget of white matter is used to support the maintenance and restoration of resting potentials and general housekeeping rather than the costs of synaptic neurotransmission. The ratio of glial cells to neurons is much higher in white matter than in gray. Schilling et al. (2022) measured the areas of the negative dip (an indicator of oxygen metabolism) at the front of the hemodynamic response functions in gray and white matter voxels and also calculated the volume fractions of tissue that are neuronal and non-neuronal, the latter being primarily composed of glial cells. Whereas the negative dip increased with increasing neuronal density in gray matter, an opposite trend was found in white matter, suggesting that the metabolic demand that produces the hemodynamic response is driven by neuronal energy requirements in gray matter but non-neuronal components (glial cells) in white matter. Therefore, reduced demand for communication could also be associated with aging-related changes in WM glial cells which have also been reported by Salas et al. (2020).

In conclusion, in the current work, we conducted a comprehensive quantification of age-related changes in BOLD profiles measured from WM on multiple spatial scales. We observed significant reductions in functional integrity in specific areas, and widespread changes in network communication as well as BOLD intensities. This work provides a unique way to characterize functional changes in the process of aging and promises to be a prelude to studies of specific disorders and pathology.

## Data availability statement

Publicly available datasets were analyzed in this study. This data can be found here: https://www.oasis-brains.org/.

## Ethics statement

The studies involving human participants were reviewed and approved by the Institutional Review Board of Washington University School of Medicine. The patients/participants provided their written informed consent to participate in this study.

## Author contributions

ML, ZD, AA, BL, and JG contributed to the conception and design of the study. ML, YG, RL, LX, YZ, and KS organized the database and developed the software. ML performed the statistical analysis and wrote the first draft of the manuscript. All authors contributed to the manuscript revision and read and approved the submitted version.
