# Supplementary material for: Changes in white matter functional networks across late adulthood
Source: Front Aging Neurosci. 2023 Jun 30;15:1204301. doi: 10.3389/fnagi.2023.1204301 (PMC10347529; doi:10.3389/fnagi.2023.1204301)
Supplement: Supplementary file 1 [file Presentation_1.PDF]

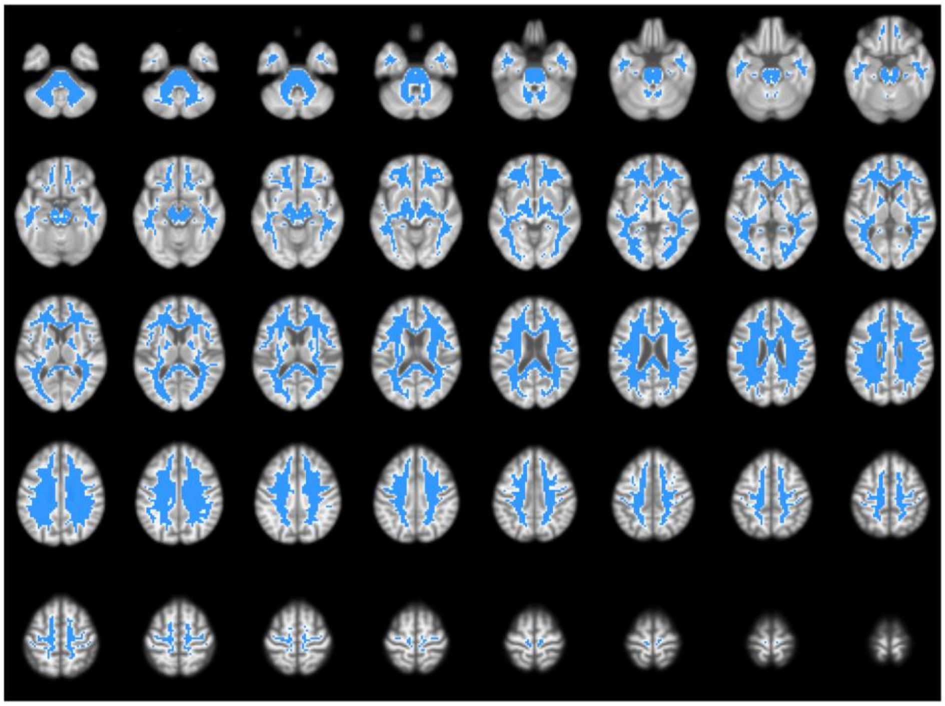

Figure S1. Visualization of the WM mask on a mean T1 in axial slices

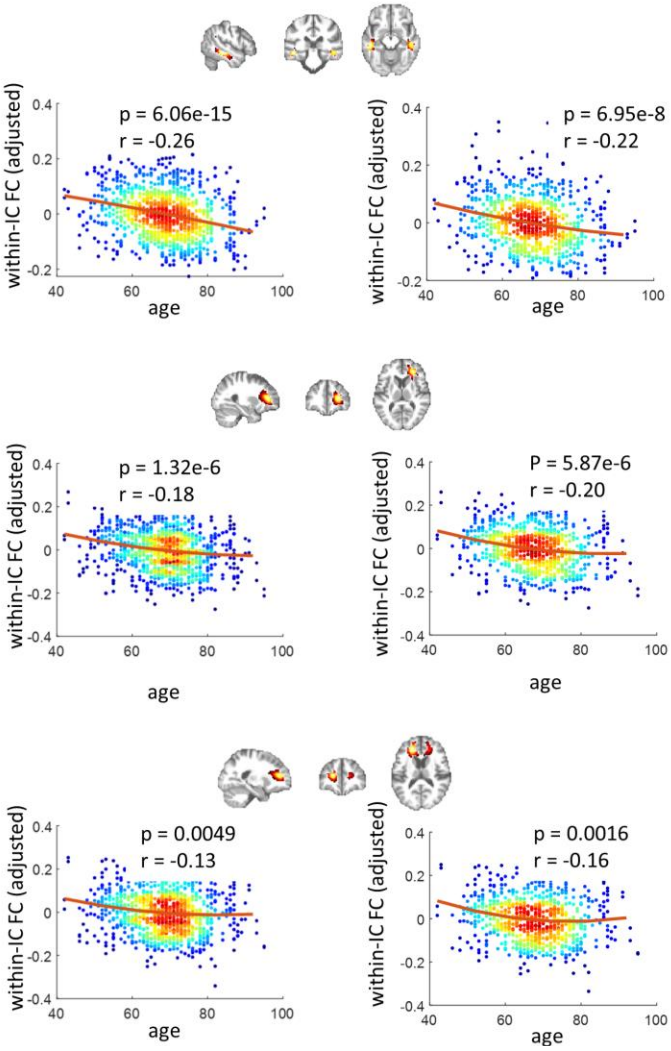

Figure S2. Relationship between within-IC FC and age based on data acquired from two different scanners (left column, replicated from Figure 2) and one of the scanners (right column)

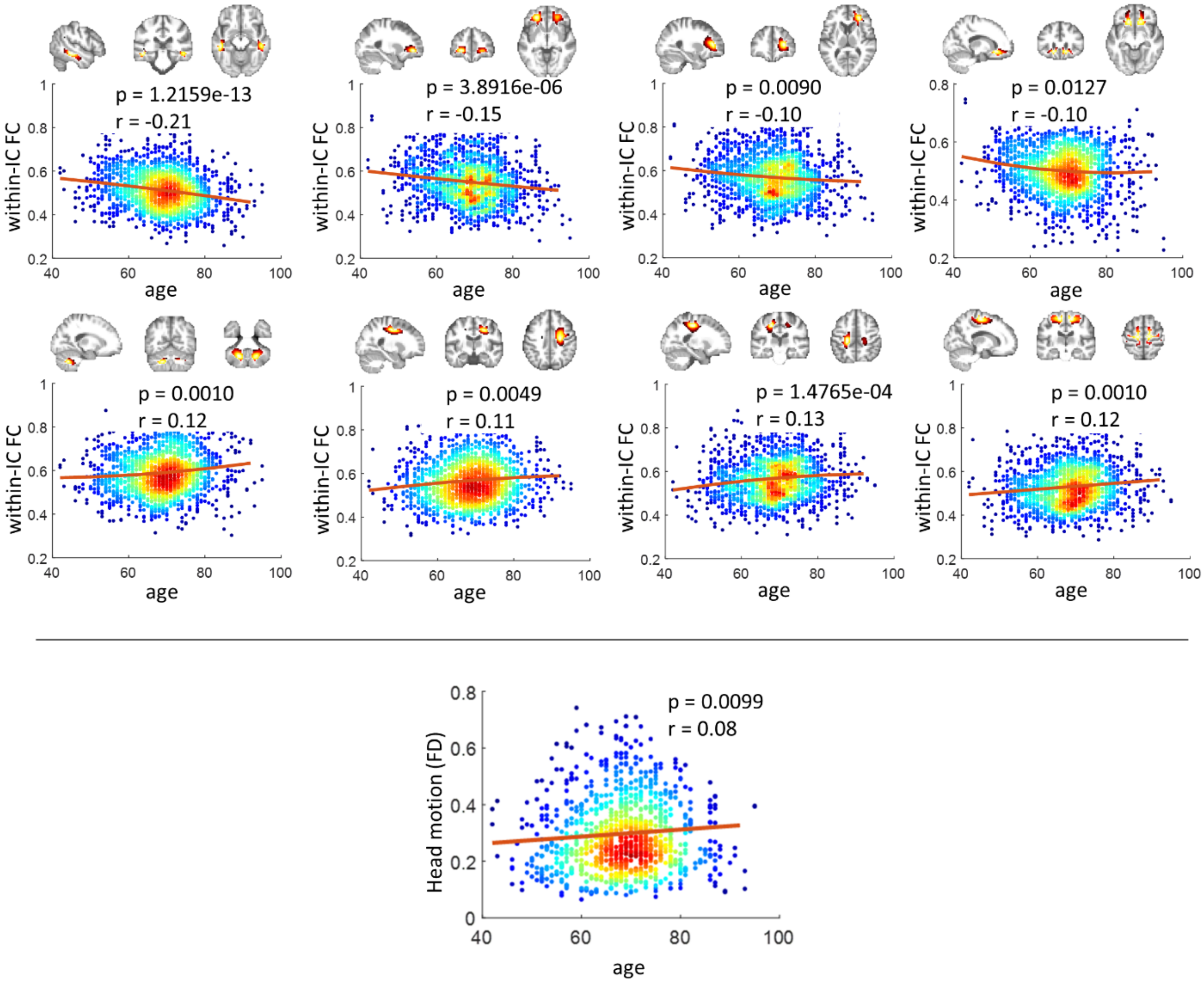

Figure S3. Relationship between within-IC FC, head motion and age. Upper panel: Eight ICs that show significant changes across age are shown ($p < 0.05$, Bonferroni correction). Each panel visualizes the spatial distribution of the IC, as well as scatters that represent the age of the subjects (x-axis) versus the within-IC FCs (y-axis). The first row corresponds to ICs that show trends toward reduced within-IC FC over age while the second row displays those with increasing trends. Lower panel: the head motion measured from the individuals are positively correlated with age.

Table S1. Bundles that correspond to the ICs.

IC1: Inferior cerebellar peduncle	IC2: Pontine crossing tract	IC3: Middle cerebellar peduncle	IC4: Middle cerebellar peduncle
IC5: Anterior corona radiata	IC6: Sagittal stratum	IC7: Middle cerebellar peduncle	IC8: Superior corona radiata
IC9: Posterior thalamic radiation	IC10: Anterior corona radiata	IC11: Superior corona radiata	IC12: Body of corpus callosum
IC13: Internal capsule	IC14: Superior corona radiata Right	IC15: Corticospinal tract	IC16: Splenium of corpus callosum
IC17: Cerebral peduncle	IC18: Superior longitudinal fasciculus Left	IC19: Anterior corona radiata	IC20: Body of corpus callosum
IC21: Anterior corona radiata Right	IC22: Superior longitudinal fasciculus Right	IC23: Splenium of corpus callosum	IC24: Anterior corona radiata
IC25: Anterior corona radiata	IC26: Superior longitudinal fasciculus Right	IC27: Sagittal stratum	IC28: Posterior thalamic radiation
IC29: Superior corona radiata	IC30: Superior longitudinal fasciculus Left	IC31: Genu of corpus callosum	IC32: Posterior corona radiata
IC33: Superior longitudinal fasciculus	IC34: Retrolenticular part of internal capsule	IC35: Superior corona radiata Left	IC36: Posterior corona radiata Right
IC37: Posterior thalamic radiation Left	IC38: Posterior thalamic radiation	IC39: Posterior corona radiata	IC40: Posterior corona radiata

Association tracts   Projection tracts   Tracts in brainstem   Commissural tracts
